# Introducing stimulogenetics, unraveling pertinent semantic ambiguity, and determining clinical relevance among novel neuromodulation strategies

**DOI:** 10.1093/biomethods/bpac019

**Published:** 2022-08-16

**Authors:** Pranjal Garg, Saidharshini Muthiah, Sumedha Sengupta

**Affiliations:** All India Institute of Medical Sciences, Rishikesh, Uttarakhand, India; Sri Muthukumaran Medical College, Hospital and Research Institute, Chennai, Tamil Nadu, India; Dr. BR Ambedkar Center for Biomedical Research, University of Delhi, Delhi, India

**Keywords:** deep brain stimulation, genetic techniques, optogenetics, chemogenetics, magnetogenetics, sonogenetics

## Abstract

Deep brain stimulation involving the stereotactic implantation of electrodes in the deeper neural tissue remains one of the most trusted nonpharmacotherapeutic approaches for neuromodulation in the clinical setting. The recent advent of techniques that can modulate the neural structure and/or function at the cellular level has stimulated the exploration of these strategies in managing neurological and psychiatric disorders. Optogenetics, which is widely employed in experimental research, is the prototype of the above techniques. Other methods such as chemogenetics, sonogenetics, and magnetogenetics have also been introduced. Although these strategies possess several noticeable differences, they have an overlapping conceptual framework enabling their classification under a singular hypernym. This article introduces this hypernym, “stimulogenetics” in an attempt to solve the pertinent ambiguity to aid the classification of existing literature. The article also compares the strategies classified under stimulogenetics and concludes that the current literature suggests that nonsurgical approaches such as chemogenetics and sonogenetics are better suited for clinical applications. However, due to the dearth of clinical studies, it is not possible to determine this definitively.

## Background

Neuromodulation is an indispensable component in the neurological disease-management toolkit. It can be defined as the targeted modification, regulation, or therapeutic alteration of central-, peripheral-, or autonomic-nervous system activity [1]. It has historically comprised direct electrical stimulation of the brain, which was later replaced by neuro-pharmacotherapeutics. However, recently, the term “brain stimulation” in clinical settings has become almost synonymous with deep brain stimulation (DBS) approaches. Brain stimulation has since evolved from a network level to the cellular level, where several approaches such as optogenetics, magnetogenetics, chemogenetics, and sonogenetics have been developed. Neuronal perturbation in these techniques is made possible by expressing genetic actuators that are sensitive to specific external stimuli [2–5]. Although these four strategies do not correspond to the same technique, similarities in their operating concept allow for a common grouping. Hence, this article introduces a hypernym, “stimulogenetics” encompassing the aforementioned strategies.

Currently, the applications of stimulogenetics are primarily restricted to basic science research. However, they possess an extraordinary potential to help re-evaluate and expand the current scope of nonpharmacotherapeutic neuromodulation in clinical applications. Hence, we also attempt to redefine the practicality of stimulogenetics in clinical settings and to provide the basis to reinvigorate clinicians’ perceptions of brain stimulation. Additionally, we assess whether the precedence of one stimulogenetic strategy over the others can be established.

## Pertinent semantic ambiguity

Semantic ambiguity in brain stimulation strategies is not unexpected as currently, out of the four aforementioned strategies, only “optogenetics” is listed as a Medical Subject Headings (MeSH) term (by the National Library of Medicine) and appears on Ontology for MIRNA Targets (OMIT) under the “genetic techniques” hypernym. MeSH and Ontology are controlled vocabularies that are extensively employed by the scientific community to represent hierarchical information. Although the omission of magnetogenetics, chemogenetics, and sonogenetics from the said vocabulary might be due to their recent addition to the neuromodulation toolkit, the lack of formal recognition of nomenclature adds ambiguity to the communication of scientific arguments.

**Figure 1:**
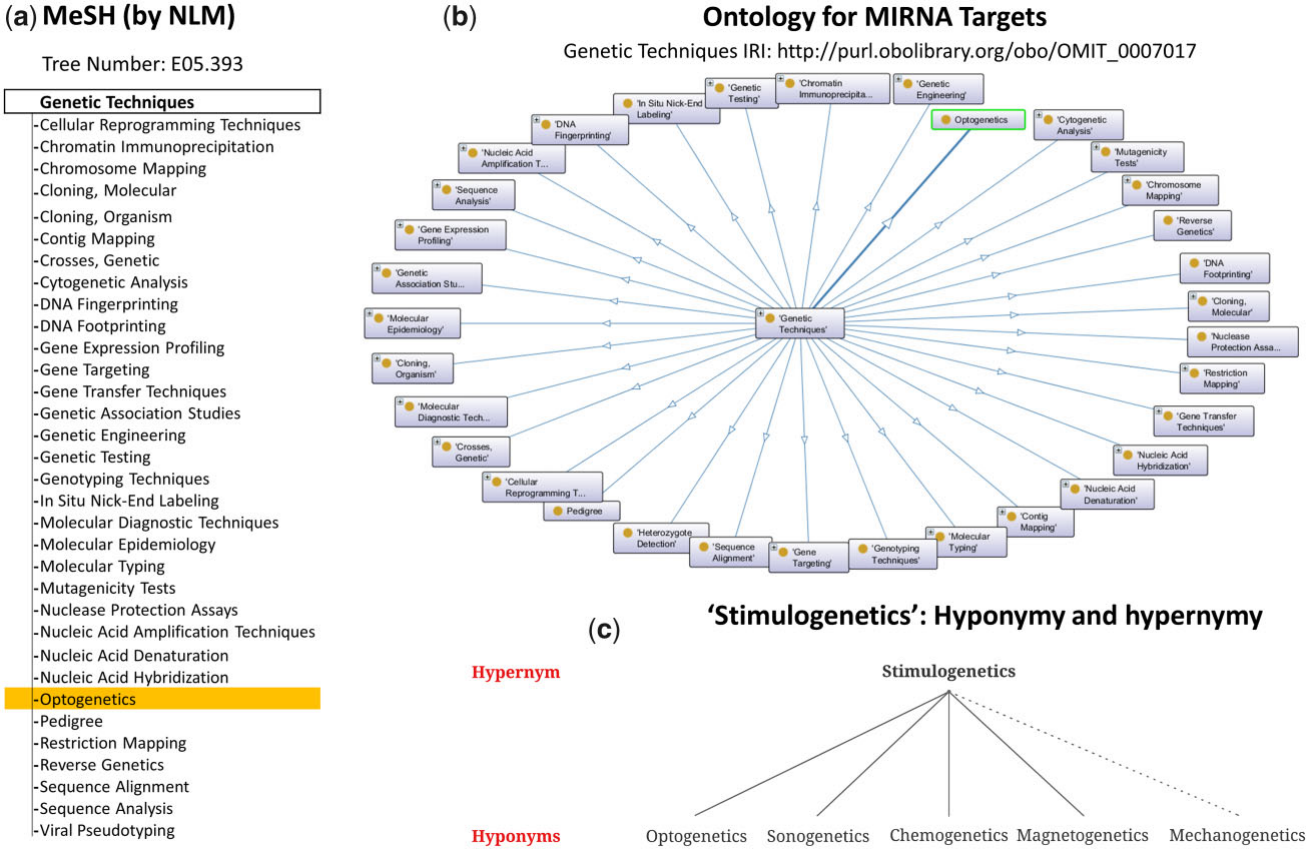
Semantics associated with novel neuromodulation strategies. **(a)** MeSH architecture with instances of the “Genetic Techniques” class. Only one of the stimulogenetic strategies, “Optogenetics,” is listed as a MeSH term (highlighted). **(b)** Visualization of “Genetic Techniques” class in OMIT. Only one of the stimulogenetic strategies, “Optogenetics,” is listed as a concept (highlighted). All the stimulogenetic strategies were queried individually in Ontology Lookup Service [8] and Ontobee [9]. The graph was constructed using Protégé [10]. **(c)** Proposed classification of neuromodulation strategies – optogenetics, sonogenetics, chemogenetics, and magnetogenetics under stimulogenetics hypernym (solid line). Due to semantic ambiguity, mechanogenetics cannot be conclusively classified under stimulogenetics (dotted line). NLM: National Library of Medicine; IRI: internationalized resource identifier.

At least one instance of this ambiguity exists in the scientific literature. Pan et al. [6] have identified a mechanosensor (Piezo1) and have developed a strategy to ultrasonically manipulate T cells labeled with the said receptors. They have termed this strategy mechanogenetics rather than sonogenetics, even though it falls within the latter definition. The ambiguity increases with the addition of a different technique termed mechanogenetics. Kim et al. [7] developed magnetoplasmonic nanoparticles which deliver mechanical stimulation to genetically labeled cells. Following the unspecified rule, associated with the etymology of the terms such as chemogenetics, sonogenetics, optogenetics, and magnetogenetics, where the type of stimulus is followed by “genetics,” this strategy was termed mechanogenetics (although naming the said strategy mechanogenetics is still debatable, as it can probably be classified under magnetogenetics). To reduce such conflicts, we advocate formalizing the associated nomenclature. As mentioned above, the addition of sonogenetics, chemogenetics, and magnetogenetics under the “genetic techniques” hypernym along with optogenetics is unwarranted. All four approaches are abundantly similar because they apparently obey the similar principle of artificial stimulation of genetically labeled cells compared to other approaches mentioned under “genetic techniques.” Consequently, there is a need to introduce a novel term encompassing these strategies. We have coined the term “stimulogenetics” to describe them. This term is constructed by combining a Latin part, “stimulo,” meaning stimulate, and an English part, “genetics.” Figure 1 illustrates the said proposal. This suggestion aims to encourage the introduction of newer, more dynamic strategies based on similar principles and to aid scientific communication. The inclusion of “stimulogenetics” as a MeSH term will allow robust indexing and will improve the discoverability of records with similar techniques. Moreover, introducing a new ontology term will aid in analyzing information, causal modeling, and maintenance of knowledgebases such as WormBase, XenBase, and FlyBase. The significant limitation encountered when the aforementioned terms are formalized is that it is quite challenging to classify strategies that employ overlapping techniques. It is plausible that newer terms with multiple prefixes will be introduced to accommodate this anomaly. Moreover, introducing the term “stimulogenetics” could provide a robust framework to tackle the said issue as the comprehensive definition of this term allows the inclusion of strategies that are difficult to classify.

## The stimulogenetics toolkit

Clinically, DBS has proven advantageous in cognitive disorders and in managing motor diseases such as Parkinson’s disease, essential tremors, etc. However, it primarily remains an invasive procedure, even though attempts have been made to develop noninvasive approaches. Challenges such as poor spatial resolution and adverse effects of unknown origin can reportedly be countered by stimulation at the microscopic level [11]. DBS can be employed to study brain circuits. However, the scope of this technique to assess the fundamental principles of neural processing and functioning is limited.

**Figure 2:**
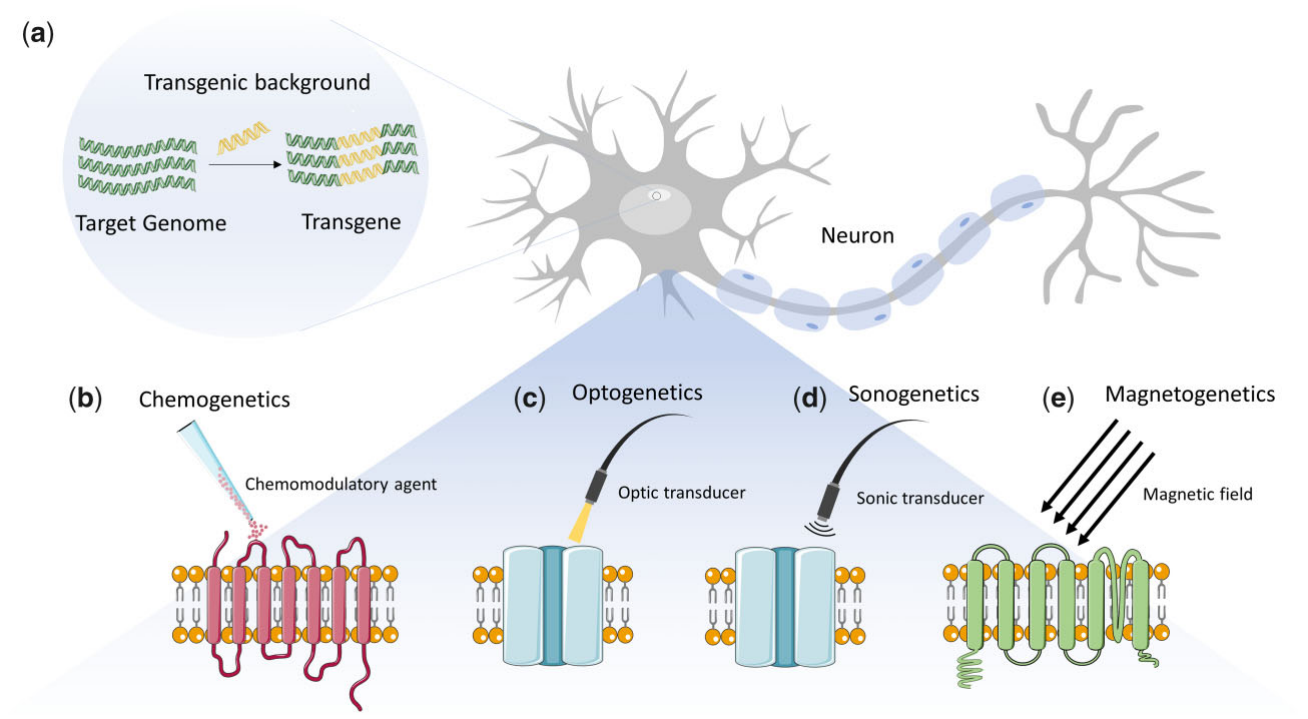
Mechanism of stimulogenetic approaches for neuromodulation. **(a)** Stimulogenetic approaches rely on the transgenic background to make the cells of interest susceptible to desirable perturbation. **(b)Chemogenetics** is small molecule mediated activation of engineered proteins. The basic principle of the technique revolves around engineering receptors such that they selectively bind to synthetic ligands. The traditional chemogenetic approaches utilize G-protein coupled receptors that were engineered to bind non-natural ligands. It exploits the fact that ion channels are suitable for manipulating the electrical properties of cells and, thus, their excitabilities [14]. **(c)Optogenetics** relies on the stimulation of light-sensitive cells rendered by synthetic opsins to modulate the cell function based on the user’s needs [2]. This strategy has been widely used in neuroscience and other systems. **(d)Sonogenetics** is the most recent stimulogenetic strategy developed by Ibsen et al. [5]. It is a noninvasive biophysical strategy that uses low-pressure ultrasonic stimuli to perturb neurons. This is accomplished by rendering cells sonic sensitive by genetically modifying them to produce ultrasound-responsive proteins/receptors. It follows similar principles of functioning as ultrasonic neural modulation. **(e)Magnetogenetics** employs magnetic stimulation to modulate cells of interest. Principally, magnetogenetics follows three mechanisms – expression of iron chaperone protein ISCA1, magneto-thermo-genetics, and torque-based methods. The commentary article by Nimpf and Keays provides an excellent brief overview of the prevalent concepts associated with magnetogenetics [13].

Stimulogenetics is theragnostic and research-friendly as it can target the neural structure or other systems with single-cell resolution. Figure 2 provides an overview of the strategies classified under stimulogenetics. In lieu of its high temporal resolution, optogenetics began the current period of neuromodulation, permitting precise control of cells on a millisecond timescale [12]. Hence, when optogenetics was introduced, it was instantly adopted by basic-science researchers but its definitive clinical adoption remains elusive. Due to the relative inaccessibility to the deep-brain neural tissue, invasive approaches have provided the elementary solution, which is also one of the most significant drawbacks of the clinical use of optogenetics [13].

On the other hand, magnetogenetics, even though it operates noninvasively, is less efficient and is slower to respond than optogenetics [13]. Sonogenetics apparently lacks the significant drawbacks of both optogenetics and magnetogenetics, and is arguably better suited for clinical settings. It also possesses a noticeable edge because ultrasonic diathermy, which exploits therapeutic analgesic heat, is already in clinical use [15]. Sonogenetics can seemingly display better spatial resolution than optogenetics and better temporal resolution than magnetogenetics and chemogenetics [12, 16]. The clinical utility of sonogenetics still requires pre-clinical studies as it is the newest among all stimulogenetic strategies, and has only recently been shown to stimulate neurons in mammals [17]. Chemogenetics has the potential to dominate the field as it can provide sustained neural excitation or inhibition for several hours with a single drug administration and is minimally invasive. Unlike other stimulogenetic strategies, it does not require specialized instruments to function. The poor temporal resolution in chemogenetics could be a boon in studying those disease processes which are independent of modulation on the millisecond time scale [18]. However, the spatial resolution of chemogenetics is poor, as the artificial ligand might modulate other genetically similar cell receptors. This can correlate the administration of chemomodulatory agents with adverse effects in chemogenetics. Nevertheless, the current primacy of optogenetics for clinical applications might possibly be superseded by sonogenetics and chemogenetics, which are arguably preferable for brain stimulation in humans over other stimulogenetic strategies. Although stimulogenetic strategies have tremendous clinical potential, several common challenges remain outstanding, such as identifying therapeutic gene products and developing transgenes and reliable gene-delivery vectors that can effectively and safely target the cells of interest. Moreover, like optogenetic tools, all stimulogenetic tools need to be nonmutagenic and nonimmunogenic [19].

## Conclusion

This article introduces the term “stimulogenetics” to characterize the strategies that follow a similar pattern of artificial modulation of genetically labeled cells. Some of the strategies classified under stimulogenetics are neuromodulating. However, we predict that their application will soon become systemic. In this discussion, we have excluded mechanogenetics from our classification of stimulogenetics due to prevailing semantic ambiguity. Finally, we have attempted to establish the precedence among stimulogenetic strategies but we hold the view that this is not absolute due to the paucity of associated clinical trials. It is plausible that each stimulogenetic strategy, with time, will be employed to manage different aspects of a disorder or different disorders entirely. Moreover, each stimulogenetic method can be individually modified to overcome the existing drawbacks and thereby widening its applicability in the research or clinical field.

## Data Availability

There are no new data associated with this article. *Conflict of interest statement.* None declared.
